# Perceptions of Rehabilitation Access After SARS-CoV-2 Infection in Romanian Patients with Chronic Diseases: A Mixed-Methods Exploratory Study

**DOI:** 10.3390/healthcare13131532

**Published:** 2025-06-27

**Authors:** Adrian Militaru, Petru Armean, Nicolae Ghita, Despina Paula Andrei

**Affiliations:** 1 “Carol Davila” University of Medicine and Pharmacy, 020021 Bucharest, Romania; adrian.militaru@drd.umfcd.ro (A.M.); petru.armean@umfcd.ro (P.A.); nicolae.ghita@drd.umfcd.ro (N.G.); 2 Clinical Department of Urology, “St. John” Clinical Hospital, 13 Vitan-Bârzești Road, Sector 4, 042122 Bucharest, Romania; 3 Clinical Department of Internal Medicine, “Prof. Dr. Theodor Burghele” Clinical Hospital, 20 Panduri Road, 061344 Bucharest, Romania; 4 Clinical Department of Neurosurgery, “Elias” University Emergency Hospital, 17 Mărăști Boulevard, 011461 Bucharest, Romania

**Keywords:** COVID-19, chronic diseases, rehabilitation services, patient satisfaction, healthcare access, mixed methods, Romania, SARS-CoV-2

## Abstract

**Background/Objectives:** The COVID-19 pandemic exposed critical vulnerabilities in healthcare systems, especially in ensuring continuity of care for patients with chronic diseases. Rehabilitation services, essential for recovery following SARS-CoV-2 infection, were among the most disrupted. This exploratory study aimed to assess Romanian patients’ perceptions of the accessibility and quality of post-COVID-19 rehabilitation services, focusing on individuals with chronic conditions. **Methods:** This exploratory cross-sectional study was conducted over a 12-month period in 2024. Data were collected from 76 adult patients diagnosed with at least one chronic condition (hypertension, diabetes mellitus, ischemic heart disease, cancer, or chronic obstructive pulmonary disease) and with confirmed prior SARS-CoV-2 infection. Most participants were recruited during outpatient specialty consultations, with a smaller number included from hospital settings, all located in Bucharest. A structured questionnaire was administered by the principal investigator after obtaining informed consent. Quantitative data were analyzed using non-parametric methods following confirmation of non-normal distribution via the Shapiro–Wilk test (*p* < 0.05). Satisfaction scores were reported as medians with interquartile ranges (IQR), and group comparisons were performed using the Mann–Whitney U test. A mixed-methods approach was employed, including thematic analysis of open-ended responses. **Results:** Patient satisfaction with rehabilitation services was consistently low. The median satisfaction scores [IQR] were accessibility 1.0 [0.0–2.0], quality of services 0.0 [0.0–4.0], staff empathy 0.0 [0.0–5.0], and perceived effectiveness 0.0 [0.0–5.0]. The median score for perceived difficulties in access was 1.0 [1.0–2.0], indicating widespread barriers. No statistically significant differences were observed between urban and rural participants or across chronic disease categories. Thematic analysis (*n* = 65) revealed key concerns including lack of publicly funded services, cost barriers, limited physician referral, service scarcity in rural areas, and demand for home-based rehabilitation options. **Conclusions:** Romanian patients with chronic illnesses and previous SARS-CoV-2 infection continue to face substantial barriers in accessing post-COVID-19 rehabilitation services. These findings highlight the need for more equitable and integrated recovery programs, especially for vulnerable populations in underserved settings.

## 1. Introduction

The COVID-19 pandemic has caused widespread disruptions in healthcare systems worldwide, prompting the reallocation of medical resources toward emergency and intensive care services. In Romania, these shifts significantly affected the continuity of care for patients with chronic illnesses, particularly by limiting access to rehabilitation services critical for recovery. Chronic conditions such as arterial hypertension, diabetes mellitus, neoplasms, chronic obstructive pulmonary disease (COPD), and ischemic heart disease are not only associated with increased risk of severe COVID-19 outcomes, but also with complex, prolonged recovery trajectories following infection [1,2].

The COVID-19 pandemic in Romania has experienced multiple waves, with over 2.8 million confirmed cases and more than 64,000 deaths reported to date (2020–2021) [3]. Patients with chronic conditions such as arterial hypertension (prevalence ~45% in the adult population), diabetes mellitus (~11.6%), and ischemic cardiovascular diseases—one of the leading causes of mortality in the country—have been disproportionately affected, presenting higher risks of severe disease and prolonged recovery periods [4]. Recent studies, such as “Imagistic Findings Using Artificial Intelligence in Vaccinated versus Unvaccinated SARS-CoV-2-Positive Patients Receiving In-Care Treatment at a Tertiary Lung Hospital” and research on “Post-COVID-19 Syndrome Based on Disease Form and Associated Comorbidities,” have highlighted complex clinical trajectories in these patient groups, emphasizing the critical need for effective and accessible rehabilitation services tailored to their specific needs [5,6].

It is important to note that not all COVID-19 patients require rehabilitation programs. The role and intensity of post-COVID-19 rehabilitation largely depend on the severity of the initial infection. Patients who experience mild or asymptomatic infections may recover fully without specialized rehabilitation, whereas those with moderate to severe disease—particularly those requiring hospitalization or intensive care—often face significant functional impairments and prolonged recovery trajectories that necessitate tailored, multidisciplinary rehabilitation interventions. This stratification ensures the efficient allocation of resources and maximizes patient outcomes by targeting rehabilitation efforts to those with the greatest needs [7,8].

Post-COVID-19 rehabilitation includes multidisciplinary interventions—such as physiotherapy, respiratory therapy, and psychological support—which are essential for restoring patients’ functional status, physical independence, and emotional well-being. International health authorities have emphasized the importance of integrating rehabilitation into long-term COVID-19 care models as an essential component of health system resilience [9,10]. Patient perceptions of rehabilitation accessibility—whether related to geographic proximity, affordability, cultural sensitivity, or provider communication—play a crucial role in shaping engagement, adherence, and overall health outcomes. When services are perceived as accessible and responsive, patients are more likely to actively participate in their recovery. Conversely, perceived barriers can lead to reduced utilization, suboptimal outcomes, and increased healthcare costs in the long term. Therefore, understanding patient perceptions is vital for designing inclusive and effective rehabilitation strategies. Although some countries have adopted structured, nationwide rehabilitation frameworks, disparities in access and quality remain evident across healthcare systems.

In Romania, the healthcare system is organized into three levels: primary care (usually provided by general practitioners), secondary care (delivered in outpatient specialty clinics), and tertiary care (hospital-based, often for complex cases). Patients with chronic diseases are primarily managed by family doctors, but diagnosis confirmation, initiation of treatment, and specialized monitoring often require access to secondary and tertiary levels of care [11].

Despite international attention to the importance of rehabilitation, there is a notable lack of empirical data from Romania assessing how patients with chronic comorbidities and COVID-19 coinfection perceive post-infection recovery services. Barriers include underdeveloped infrastructure, uneven coverage from the National Health Insurance House, and major geographic disparities. In particular, rural communities may face disproportionate challenges in accessing even basic rehabilitation support.

Furthermore, identifying eligible patients for this study proved difficult. The inclusion criteria—requiring a confirmed SARS-CoV-2 infection combined with at least one of the five specified chronic conditions—greatly limited the available population due to the elevated mortality among these vulnerable groups during the pandemic. Over a 12-month period, 76 patients were identified and included in the study.

All interviews were conducted by the principal investigator, in person or by phone, after obtaining written or verbal informed consent. To ensure accuracy, interviews were recorded and transcribed for analysis. The questionnaire used in this research was specifically developed by the study team, based on clinical relevance and contextual needs, as no validated tool addressing post-COVID-19 rehabilitation for patients with chronic diseases in Romania was available at the time.

This exploratory study aims to assess the perceived quality and accessibility of post-COVID-19 rehabilitation services among Romanian patients with chronic illnesses and confirmed SARS-CoV-2 infection. Additionally, it explores how comorbidities and place of residence may influence patient satisfaction and perceived barriers to recovery, with the goal of informing inclusive and equitable rehabilitation policy development.

## 2. Materials and Methods

### 2.1. Study Design and Participants

This exploratory cross-sectional study was conducted over a 12-month period in 2024 in Bucharest, Romania. The target population included adult patients (≥18 years) diagnosed with at least one of the following chronic illnesses: arterial hypertension, diabetes mellitus, ischemic heart disease, neoplasms, or chronic obstructive pulmonary disease (COPD), all of whom had also experienced a confirmed SARS-CoV-2 infection and had used rehabilitation services following the acute phase of COVID-19.

Inclusion criteria were (1) age ≥ 18 years; (2) documented diagnosis of at least one of the five chronic conditions listed above; (3) confirmed SARS-CoV-2 infection by PCR or antigen testing; (4) self-reported engagement in rehabilitation services after the COVID-19 episode; and (5) ability to provide informed consent.

Exclusion criteria included (1) patients with severe cognitive impairment or mental illness that would prevent meaningful participation in the interview; (2) patients who did not engage in any form of rehabilitation post-COVID-19; and (3) refusal or inability to provide informed consent.

Eligible patients were identified during specialist outpatient consultations or hospital follow-up visits. The selection process was based entirely on voluntary participation, without coercion or incentives. Physicians informed potentially eligible patients about the study and referred them to the principal investigator, who confirmed eligibility, obtained verbal or written informed consent, and administered the questionnaire. No patients were pre-screened using electronic records or administrative databases. Participation was open to all who met the criteria and agreed to take part in the interview. All procedures adhered to the principles outlined in the Declaration of Helsinki [12].

### 2.2. Data Collection

Data were collected using a structured questionnaire specifically developed by the research team for this study. The instrument was not previously validated but was informed by the study objectives and prior literature on rehabilitation accessibility and health service evaluation [13,14]. The final questionnaire is included as Supplementary Material S1.

The questionnaire combined closed-ended items rated on a five-point Likert scale (1 = very poor to 5 = very good) with open-ended questions capturing patient experiences, perceived service quality, and barriers to rehabilitation. Topics addressed included the type and timing of rehabilitation, delivery setting (public or private), professional communication, and logistical accessibility.

All interviews were conducted by the principal investigator, either face-to-face or by telephone, based on participant availability. Interviews were audio-recorded with prior consent and transcribed verbatim to ensure accuracy.

Data were collected using an 18-item questionnaire developed specifically for this study by the research team. The instrument employed a mixed-methods structure, including 17 closed-ended questions rated on Likert scales and a single open-ended item designed to capture personal perceptions and suggestions regarding rehabilitation services. The questionnaire investigated the following key domains:

- Accessibility of rehabilitation services (timing, location, availability)
- Type and setting of services received (public vs. private; inpatient vs. outpatient)
- Perceived quality of medical staff and service delivery
- Effectiveness of rehabilitation in improving overall health
- Barriers to accessing services (geographical, financial, procedural)

Participants were asked to evaluate their experiences with rehabilitation services following COVID-19 without being provided with a standardized definition. The questionnaire aimed to capture their personal understanding and perception of any form of rehabilitation they had encountered, whether formal or informal

The questionnaire was administered by the principal investigator, either face-to-face or via telephone, after obtaining written or verbal informed consent. All interviews were conducted in Romanian and were audio-recorded to ensure accuracy. Transcripts were anonymized and subsequently entered into a Microsoft Excel database for analysis.

Although the study sample comprised 76 participants, which may be considered limited in broader epidemiological research, this size is acceptable and methodologically appropriate for exploratory designs. Similar mixed-methods studies on healthcare access or patient perceptions have employed comparable sample sizes to generate context-specific insights and identify initial patterns that warrant further investigation [15,16]. Given the strict inclusion criteria—requiring both confirmed SARS-CoV-2 infection and the presence of at least one chronic condition from a predefined list—the recruitment process was inherently restrictive. Additionally, the elevated COVID-19-related mortality among chronically ill populations in Romania further reduced the available participant pool. In this context, the study’s sample is consistent with published exploratory research in similar healthcare settings [17].

### 2.3. Data Analysis

In addition to the 17 closed-ended items, the questionnaire included one open-ended question inviting participants to share any additional comments or suggestions regarding rehabilitation services. Although the question was broad and non-directive, many respondents spontaneously described specific experiences, barriers, and unmet needs that were not directly addressed in the structured items. These narrative responses were transcribed and analyzed thematically, enabling the identification of recurring patterns related to service availability, costs, referral gaps, and preferences for home-based care. This qualitative input formed the basis for the themes presented in Section 3.

Quantitative data were analyzed using descriptive statistics, including medians, interquartile ranges (IQR), and frequencies. The normality of distribution for ordinal satisfaction scores was assessed using the Shapiro–Wilk test. As most variables deviated from normality (*p* < 0.05), non-parametric methods were considered appropriate. Based on these findings, group comparisons—such as those between urban and rural residents or between chronic disease categories—were conducted using the Mann–Whitney U test for ordinal variables. Categorical variables were analyzed using Fisher’s exact test or the chi-square test, depending on the number of categories. The Shapiro–Wilk test results were used solely to guide the choice of statistical methods and were not reported in the Results section, as they were not central to the research objectives. A *p*-value < 0.05 was considered statistically significant.

Qualitative responses were analyzed thematically. Two researchers independently coded transcripts and identified recurring patterns related to satisfaction, accessibility, and structural barriers. Coding discrepancies were resolved through discussion and consensus.

### 2.4. Ethical Considerations

The study was conducted in accordance with the Declaration of Helsinki [12] and applicable Romanian legislation, including Law No. 190/2018 and the EU General Data Protection Regulation (GDPR) 2016/679. Of the 76 participants, 53 provided written informed consent during in-person interviews, while 23 participants were interviewed by telephone and initially gave verbal consent, following a full explanation of the study’s objectives, procedures, confidentiality terms, and voluntary nature. These verbal consents were documented through audio recordings made at the time of the interview. Additionally, participants who provided verbal consent were offered the option to receive a written consent form by post or email, which they could sign and return if they wished. Several participants requested and received the written form for their records. This procedure was reviewed and approved by the Ethics Committee of Prof. Dr. Theodor Burghele Clinical Hospital (Approval No. 4/12.10.2020). Participant confidentiality and anonymity were strictly upheld, with no identifying information collected beyond optional email addresses, which were securely encoded.

### 2.5. Use of Artificial Intelligence Tools

No generative artificial intelligence (GenAI) tools were used to generate or analyze any data in this study. Minor language editing assistance was provided using AI-based tools; however, all final content was manually reviewed and edited by the authors, who take full responsibility for the accuracy and integrity of the manuscript.

## 3. Results

### 3.1. Characteristics of the Study Sample

The final study sample consisted of 76 patients diagnosed with one or more chronic conditions and with confirmed prior SARS-CoV-2 infection. The mean age was 53.8 years (±14.8), with 64.5% *(n* = 49) of participants under 60 years of age and 35.5% (*n* = 27) aged 60 or older. A slight majority were female (53.9%, *n* = 41), and 61.8% (*n* = 47) resided in urban areas.

Regarding educational background, 55.3% (*n* = 42) had completed high school, while 31.6% (*n* = 24) had pursued higher education. Most participants (97.4%) had at least one chronic condition. Stratified by comorbidity count, 48.7% (*n* = 37) had 2–3 chronic illnesses, and another 48.7% (*n* = 37) had a single chronic condition; only 1.3% (*n* = 1) reported four or more.

COVID-19 clinical history revealed that the vast majority (97.4%, *n* = 74) had contracted the virus more than 12 months before the interview. The most frequently reported severity level was mild (47.4%, *n* = 36), with a smaller proportion experiencing critical illness (2.6%, *n* = 2). Regarding acute care, 35.5% (*n* = 27) reported being hospitalized for COVID-19, while the remaining 64.5% (*n* = 49) managed their illness on an outpatient basis.

This diverse profile—spanning multiple comorbidities, age categories, and COVID-19 experiences—supports the exploratory nature of the study and provides a meaningful foundation for assessing perceived access to rehabilitation services.

To provide additional insight into how sociodemographic and clinical characteristics may differ by place of residence, Table 1 presents a combined overview, including both pooled data and a stratified analysis comparing participants from urban and rural areas. Urban respondents were more likely to be aged ≥ 60 (*p* = 0.0273) and to have attained higher levels of education (*p* = 0.0390). No statistically significant differences were observed in sex distribution, number of chronic conditions, or hospitalization history due to COVID-19. These findings provide an important contextual background for interpreting perceptual differences related to rehabilitation access, which are further explored in the following section.

These observed differences were statistically tested, and the results are summarized in Table 2.

### 3.2. Satisfaction with Post-COVID-19 Rehabilitation Services

#### Interpretation of Satisfaction Scores (*n* = 76)

1. Accessibility (0.97 ± 1.02) (question 13):
  This extremely low average score indicates that the majority of patients perceived post-COVID-19 rehabilitation services as very difficult to access. Barriers likely included a lack of nearby facilities, limited state-covered options, and poor referral practices. The relatively high standard deviation suggests that experiences varied significantly among respondents, possibly based on location or care setting.
2. Quality of Services (1.64 ± 2.14) ( question 14)
  Patients rated the overall quality of rehabilitation services as poor, with an average score below 2. This low satisfaction may reflect deficiencies in infrastructure, availability of equipment, or fragmentation of services. The high standard deviation implies that while a small subset of patients may have had satisfactory experiences, the majority were clearly dissatisfied.
3. Staff Empathy (1.71 ± 2.21) (question 15)
  This score highlights a general perception of insufficient empathy and preparedness among healthcare professionals involved in rehabilitation. The result may point to limited patient-provider communication, lack of individualized care, or overburdened staff. The wide variation again suggests heterogeneous experiences.
4. Perceived Effectiveness (1.76 ± 2.27) (question 16)
  Patients reported that rehabilitation interventions had limited impact on improving their overall health status. This may be linked to delayed access, incomplete recovery protocols, or inconsistent follow-up. The score reflects a lack of perceived therapeutic benefit, despite the recognized importance of rehabilitation in post-COVID care.
5. Difficulties in Access (1.37 ± 0.78) (question 17)
  Although this item is inversely scored (lower values indicate fewer difficulties), the result still reflects substantial access-related challenges. Patients frequently encountered issues such as long waiting times, high costs, or a lack of available services within their geographic region. The relatively lower standard deviation indicates that these difficulties were consistently experienced across the sample.

A comprehensive summary of the mean satisfaction scores, including standard deviations, for each evaluated domain is shown in Table 3**.**

Overall, the results indicate a markedly low level of satisfaction with post-COVID-19 rehabilitation services among patients with chronic illnesses. Accessibility received the lowest average score, reflecting systemic barriers that limited patients’ ability to engage with rehabilitation programs. Similarly, ratings for service quality, staff empathy, and perceived effectiveness were all notably low, suggesting widespread dissatisfaction across multiple domains of care. These findings point to major structural and organizational deficiencies in the delivery of rehabilitation services during the pandemic recovery phase in Romania.

### 3.3. Subgroup Comparisons

#### Interpretation of Urban–Rural Differences

1. Accessibility (Question 13)

Urban: 1.0 [0.0–2.0]|Rural: 1.0 [0.0–1.0]

Both groups rated accessibility poorly, with rural respondents showing a more compressed distribution. This suggests that barriers to accessing rehabilitation services remain prevalent across Romania, especially in rural areas with limited infrastructure.

2. Quality of Services (question 14)

Urban: 0.0 [0.0–4.0]|Rural: 0.0 [0.0–4.0]

Median satisfaction with service quality was null in both groups, highlighting a widespread perception of very low-quality rehabilitation services. While the interquartile range suggests some isolated higher ratings, the general trend reflects severe dissatisfaction.

3. Staff Empathy( question 15)

Urban: 0.0 [0.0–4.0]|Rural: 0.0 [0.0–5.0]

Median scores for empathy were also 0, with only a few rural respondents reporting better interpersonal care. The findings indicate that for most patients, empathy and human connection in care delivery were severely lacking.

4. Perceived Effectiveness(question 16)

Urban: 0.0 [0.0–4.0]|Rural: 0.0 [0.0–5.0]

Across both residence groups, patients reported minimal perceived health improvement from rehabilitation, as shown by the zero median. This underlines a major disconnect between service provision and patient expectations or outcomes.

5. Difficulties in Access (question 17)

Urban: 1.0 [1.0–2.0]|Rural: 1.0 [1.0–1.0]

Although both groups reported difficulty, rural patients showed less variability and slightly worse scores, suggesting more consistent and entrenched access problems in peripheral regions.

These urban–rural subgroup results are summarized in Table 4, which presents the median scores, interquartile ranges, and statistical comparisons for each evaluation item.

Subgroup analysis revealed uniformly low patient satisfaction across all dimensions, with median scores of 0 for quality, empathy, and perceived effectiveness. These findings reflect widespread disappointment and systemic underperformance. While rural respondents reported slightly narrower ranges and greater access difficulties, differences did not reach statistical significance. The consistent dissatisfaction signals a critical need to improve rehabilitation experiences and outcomes nationwide, regardless of location.

### 3.4. Disease-Specific Results

#### Subgroup Comparisons

Patients with cancer reported substantially higher satisfaction across all rehabilitation domains, with median scores reaching 4 or 5 in quality, empathy, and perceived effectiveness. This may reflect more individualized care or access to private/oncology-specific services.

A detailed comparison of satisfaction scores across chronic disease categories is presented in Table 5.

In contrast, participants with hypertension, diabetes, COPD, or ischemic heart disease showed very low satisfaction, with median scores of 0 for most items, suggesting systemic underperformance in addressing their rehabilitation needs.

Despite shared access difficulties across all groups (median = 1), cancer patients also reported slightly more consistent access. These differences underscore possible disparities in service quality by disease type, possibly linked to care specialization or funding structures.

### 3.5. Qualitative Analysis

Out of 76 participants, 76 provided answers to the open-ended question included in the survey. However, only 65 responses contained relevant and analyzable content that could be categorized into thematic areas. The remaining 11 responses were either too vague (e.g., “nothing to add”, “no comment”, or “I don’t know”) or lacked specific reference to rehabilitation services and therefore were excluded from thematic coding.

The main themes identified from the 65 relevant qualitative responses are summarized in Table 6**.**

Thematic analysis of the 65 relevant responses revealed five primary concerns related to the accessibility and quality of post-COVID-19 rehabilitation services:

1. Cost-related barriers were the most frequently cited issue. Many respondents emphasized the high financial burden of private rehabilitation services and called for better state-funded coverage or reimbursement mechanisms.
2. Lack of public services and coverage emerged as a major theme, particularly the limited number of rehabilitation facilities affiliated with the public health system.
3. Insufficient referral and lack of information were highlighted by participants who reported never being advised by their treating physicians about the availability or importance of post-COVID-19 rehabilitation.
4. Availability in rural areas was a persistent concern. Respondents from rural regions reported significant geographic disparities in access to rehabilitation, citing long travel distances and service scarcity.
5. Home-based service needs were expressed by several patients who advocated for in-home rehabilitation as a more accessible and affordable alternative.

These qualitative findings reinforce the quantitative results, highlighting systemic inequities and unmet needs in Romania’s post-COVID-19 rehabilitation infrastructure. They also suggest a need for more targeted policies, especially for underserved populations in rural settings.

## 4. Discussion

### 4.1. Interpretation of Findings

The results of this study reveal a persistently low level of satisfaction with post-COVID-19 rehabilitation services among Romanian patients with chronic illnesses, regardless of their geographic or diagnostic profile. These conclusions were supported by non-parametric subgroup analyses using the Mann–Whitney U test, which revealed no statistically significant differences between urban and rural groups or between chronic disease categories. The lack of statistically significant differences between urban and rural groups suggests that these shortcomings are systemic rather than region-specific. Similarly, no meaningful variation in satisfaction was observed across chronic disease subgroups, indicating the presence of structural healthcare barriers that affect patients uniformly.

Participants commonly cited inadequate service availability, financial barriers, and the absence of individualized, sustained recovery programs. These concerns align with findings from other healthcare systems. For instance, post-pandemic assessments in the United Kingdom identified severe regional disparities in rehabilitation access, especially during COVID-19 surges [18]. In the United States, access to rehabilitation remains heavily influenced by insurance coverage, deepening existing socioeconomic disparities [19].

In Southern and Eastern Europe, structural inefficiencies have also been documented. Italian studies have pointed to poor integration between primary care and rehabilitation services, leading to fragmented patient experiences and diminished continuity of care [20]. Additionally, post-pandemic evaluations in countries such as Spain have documented a significant increase in workload for rehabilitation services, with many hospitals initiating specific outpatient follow-up consultations for COVID-19 patients [21]. In Germany, qualitative interviews with general practitioners revealed the challenges of managing long COVID cases in primary care, highlighting the need for integrated referral pathways to rehabilitation [22]. These findings underscore the uneven regional implementation of rehabilitation strategies across Europe and support the need for enhanced structural planning in Central and Eastern Europe.

Central and Eastern European countries, including Romania, share similar limitations, such as under-resourced outpatient programs and low prioritization of rehabilitation in national health agendas [20].

Despite these constraints, substantial evidence supports the clinical and economic benefits of rehabilitation in post-COVID and chronic disease management. Structured physical therapy programs have demonstrated improvements in respiratory function, fatigue, and quality of life [6]. Furthermore, integrating psychological support into rehabilitation is associated with better long-term outcomes, particularly among patients with pre-existing chronic conditions and post-viral syndromes [10,23]. However, mental health interventions remain underutilized in the rehabilitation landscape of many low- and middle-income countries.

### 4.2. Policy Implications

The study’s findings highlight a pressing need for coordinated health policy responses to reduce inequities in rehabilitation access and quality. Several concrete measures are recommended:

- Expand state-funded outpatient rehabilitation services, especially in rural and underserved regions, to reduce geographic disparities.
- Integrate rehabilitation into national chronic disease strategies, ensuring follow-up care for multimorbid patients recovering from COVID-19.
- Increase the availability of home-based rehabilitation options, including the use of digital platforms and community health networks.
- Establish systematic referral protocols, requiring physicians to assess rehabilitation needs during post-acute care consultations.
- Embed psychological support and nutritional counseling into all standard rehabilitation packages to promote holistic recovery.

These recommendations are aligned with emerging evidence from other countries. A recent qualitative study conducted in Germany explored patient and provider experiences with a multimodal, home-based rehabilitation program delivered via telemedicine for post-acute COVID-19 patients. The program was perceived as accessible, effective, and less burdensome compared to traditional center-based care [24]. Such remote rehabilitation models could address key barriers reported by Romanian patients in our study, particularly those related to travel distance, limited service availability, and mobility challenges. Therefore, integrating telerehabilitation into national frameworks may offer a scalable and cost-effective policy solution.

Implementation of these strategies will require cross-sector collaboration and increased investment in rehabilitation infrastructure. In the context of an aging population and a high burden of chronic illness, such reforms are essential to improve health outcomes and reduce long-term healthcare costs [11,20].

### 4.3. Strengths

This study offers a unique contribution to the limited body of evidence on post-COVID-19 rehabilitation in Central and Eastern Europe. A major strength lies in its mixed-methods approach, which combined structured quantitative items with open-ended questions to capture both measurable satisfaction indicators and in-depth personal experiences.

Unlike many studies that rely exclusively on self-administered surveys, all data in this study were collected by the principal investigator through direct interviews—either face-to-face or via telephone—ensuring a high level of consistency, completeness, and clarity in responses. This approach also allowed participants to elaborate on their perceptions and barriers in a conversational format.

The inclusion of patients with diverse chronic conditions—hypertension, diabetes mellitus, ischemic heart disease, cancer, and COPD—across both urban and rural settings improves the transferability of findings and supports a broader understanding of the systemic challenges affecting multimorbid populations in the post-pandemic phase.

### 4.4. Limitations

Despite its strengths, this study is subject to several limitations. First, although the final sample size (*n* = 76) is larger than that of previous pilot studies on similar topics, it remains relatively small compared to the total number of Romanian patients affected by chronic illness and COVID-19. However, this limitation is largely explained by the strict eligibility criteria applied. Participants were required to have both a documented chronic condition and a confirmed SARS-CoV-2 infection, followed by engagement in rehabilitation services. The overlap of these factors proved to be uncommon, particularly due to the high mortality risk associated with these chronic diseases, even in the absence of COVID-19. The addition of a severe viral coinfection substantially reduced the number of potential survivors eligible for post-acute follow-up and rehabilitation. This reflects not a sampling bias, but a genuine demographic and clinical constraint. Nevertheless, similar sample sizes have been used in previous exploratory studies addressing post-COVID rehabilitation in hospitalized or chronically ill populations [6,7].

Secondly, for ethical and confidentiality reasons, we did not collect or report detailed information regarding the specific counties from which participants were recruited. This decision was made to preserve respondent anonymity, especially in under-resourced regions where service availability may be limited. Revealing such data could unintentionally lead to geographic stigmatization or bias in interpreting healthcare disparities. Moreover, mapping and evaluating rehabilitation services at the county level was beyond the scope of this exploratory study, but may represent an important direction for future research.

Third, the cross-sectional design limits causal inference. The study captures patient perceptions at a single point in time, without accounting for changes that may occur throughout the rehabilitation trajectory or due to evolving healthcare policy.

Although all data were collected in medical facilities located in Bucharest, the Romanian capital, participants originated from various regions of the country. No geographic restrictions were imposed during recruitment. As a national referral hub with several university-affiliated centers, Bucharest frequently receives complex or difficult-to-manage cases referred from across Romania, including rural and underserved areas. Therefore, while the findings are not based on a randomized national sample, they may still capture a heterogeneous range of patient experiences and challenges relevant to broader healthcare access patterns.

Fourth, while interviews were conducted by a trained investigator to ensure data accuracy and completeness, responses remain inherently subjective and may be influenced by interpretation or recall bias.

Moreover, the use of both face-to-face and telephone interviews may have introduced additional bias, including social desirability, recall, or interviewer-related effects. Differences in communication dynamics between these modalities could influence how patients understood and responded to questions, particularly in sensitive areas such as rehabilitation needs or healthcare experiences. Additionally, the assessment of COVID-19 illness severity relied on patient self-report rather than clinical records or standardized scales, which may affect the consistency and clinical accuracy of this variable.

Finally, this study focuses solely on the patient’s perspective. Additionally, given the use of ordinal satisfaction items, we employed non-parametric statistical methods for subgroup comparisons. While appropriate for the data type, this approach does not estimate effect size magnitudes and may limit interpretability. Future research would benefit from integrating the views of healthcare professionals, therapists, and administrators to better understand system-level challenges and facilitate more comprehensive reforms. Another limitation is the lack of a standardized definition of ‘rehabilitation’ provided to participants. As a result, responses may reflect varied individual interpretations, including but not limited to physical, respiratory, psychological, or occupational therapy. This heterogeneity may have influenced the consistency and comparability of satisfaction ratings.

### 4.5. Future Directions

Building on the current findings, future research should aim to conduct larger-scale, longitudinal studies to evaluate the effectiveness and accessibility of post-COVID-19 rehabilitation programs in diverse settings. Special attention should be given to rural populations, elderly patients, and those with multiple comorbidities. Additionally, pilot programs exploring the integration of telerehabilitation and home-based services within Romania’s public healthcare system would offer valuable insights into feasibility and patient outcomes. Cross-disciplinary studies involving healthcare providers, policy-makers, and patients could further identify barriers and facilitators to implementing sustainable rehabilitation strategies at the national level. Additionally, future studies should consider including variables such as travel time, transportation methods, and out-of-pocket expenses related to rehabilitation access. These factors may significantly impact participation, especially for patients living in remote or underserved areas, and are essential for designing equitable and accessible service models. Establishing a national registry of post-COVID rehabilitation needs and outcomes may also support evidence-based planning and resource allocation.

## 5. Conclusions

This exploratory study highlights a consistent pattern of dissatisfaction among Romanian patients with chronic diseases regarding post-COVID-19 rehabilitation services. The findings reveal uniformly low satisfaction scores across all key domains—accessibility, service quality, staff empathy, and perceived effectiveness—irrespective of geographic location or specific comorbidity.

Non-parametric analyses using the Mann–Whitney U test confirmed the absence of statistically significant differences between urban and rural groups or among chronic disease categories. This reinforces the interpretation that the barriers identified are systemic and structural, not isolated or population-specific.

Qualitative responses further underscored patient concerns related to limited public service availability, financial barriers, insufficient referral practices, and the lack of individualized care. These issues reflect long-standing weaknesses in Romania’s rehabilitation infrastructure, which were further exacerbated by the COVID-19 pandemic.

By documenting both the extent and nature of these barriers, the study contributes to a better understanding of the post-pandemic rehabilitation needs of chronically ill patients. These insights can inform the development of more equitable, integrated, and patient-centered rehabilitation models in Romania and other under-resourced healthcare systems.

Addressing the identified structural gaps through coordinated policy reforms may significantly improve long-term outcomes for post-COVID-19 patients and reduce the burden on healthcare systems coping with chronic multimorbidity.

## Figures and Tables

**Table 1:** Demographic and clinical characteristics of the study sample, stratified by area of residence (*n* = 76).

Variable	Category	Urban (*n* = 47)	Rural (*n* = 29)	Total (*n* = 76)
Age group	<60	35	14	49
Age group	≥60	12	15	27
Sex	Female	28	13	41
Sex	Male	19	16	35
Education level	High school	26	16	42
Education level	Higher education	18	6	24
Education level	Primary	1	0	1
Education level	Secondary	2	7	9
Chronic conditions	None	0	0	0
Chronic conditions	1	22	15	37
Chronic conditions	2–3	23	14	37
Chronic conditions	≥4	2	0	2
COVID hospitalization	No	30	23	53
COVID hospitalization	Yes	17	6	23
COVID severity	Mild	23	13	36
COVID severity	Moderate	21	13	34
COVID severity	Severe	2	2	4
COVID severity	Critical	1	1	2
Time since COVID	3–6 months	1	0	1
Time since COVID	6–12 months	0	1	1
Time since COVID	>12 months	46	28	74

Legend: Distribution of study participants (*n* = 76) by demographic, educational, clinical, and COVID-19-related characteristics, stratified by place of residence (urban vs. rural). Values represent the number of participants in each category, with totals shown for the pooled sample. Chronic conditions refer to hypertension, diabetes mellitus, ischemic heart disease, neoplasm, and chronic obstructive pulmonary disease (COPD). COVID-19-related variables include hospitalization status, illness severity, and time elapsed since confirmed SARS-CoV-2 infection. Abbreviation: COPD = chronic obstructive pulmonary disease.

**Table 2:** Statistical comparison of baseline characteristics by place of residence (urban vs. rural).

Variable	Test	*p*-Value
Age group	Fisher’s exact test	0.0273
Sex	Fisher’s exact test	0.2425
Education level	Chi-squared test	0.0390
Chronic group	Chi-squared test	0.5156
COVID hospitalization	Fisher’s exact test	0.2017
COVID severity	Chi-squared test	0.9360
Time since COVID	Chi-squared test	0.3261

Legend: Statistical comparison of baseline characteristics between urban and rural participants. Tests used include Fisher’s exact test for binary variables and Chi-squared test for multi-category variables. *p*-values indicate the significance level of differences between the two groups for each variable assessed.

**Table 3:** Mean Scores on Satisfaction Items.

Evaluation Item	Mean Score (±SD)
Accessibility	0.97 ± 1.02
Quality of services	1.64 ± 2.14
Staff empathy	1.71 ± 2.21
Perceived effectiveness	1.76 ± 2.27
Difficulties in access	1.37 ± 0.78

Legend: Mean satisfaction scores (±standard deviation) for five domains of post-COVID-19 rehabilitation, based on responses from 76 participants. Items were rated on a 5-point Likert scale ranging from 1 (very poor) to 5 (very good). Lower scores indicate lower satisfaction. For the item “Difficulties in access”, lower scores reflect fewer perceived barriers.

**Table 4:** Comparison of Patient-Reported Outcomes on Rehabilitation Services Between Urban and Rural Respondents (Median [IQR]).

Evaluation Item	Urban (Median [IQR])	Rural (Median [IQR])	Mann–Whitney U *p*-Value
Satisfaction with accessibility of recovery services (1 = very poor 5 = very good)	1.0 [0.0–2.0]	1.0 [0.0–1.0]	0.9684
Satisfaction with the quality of recovery services received (1–5)	0.0 [0.0–4.0]	0.0 [0.0–4.0]	0.9078
Empathy and training of medical staff (1–5)	0.0 [0.0–4.0]	0.0 [0.0–5.0]	0.8931
Recovery contributed to improving overall health (1–5)	0.0 [0.0–4.0]	0.0 [0.0–5.0]	0.8878
Difficulties accessing services (1–5)	1.0 [1.0–2.0]	1.0 [1.0–1.0]	0.1249

Legend: Comparison of median scores and interquartile ranges (IQR) for key evaluation items between urban and rural participants. Data are presented as median [IQR]. Statistical comparisons were performed using the Mann–Whitney U test, appropriate for non-normally distributed ordinal data. None of the observed differences reached statistical significance (*p* > 0.05 for all comparisons).

**Table 5:** Patient-Reported Rehabilitation Experience by Primary Chronic Condition (Median [IQR]).

Chronic Condition	Accessibility	Quality of Services	Staff Empathy	Perceived Effectiveness	Difficulties in Access
COPD	1.0 [0.0–1.0]	0.0 [0.0–4.0]	0.0 [0.0–4.25]	0.0 [0.0–4.25]	1.0 [1.0–1.25]
Cancer	1.0 [1.0–1.0]	4.0 [3.0–5.0]	4.0 [4.0–4.0]	5.0 [4.0–5.0]	1.0 [1.0–1.0]
Diabetes	1.0 [0.0–1.5]	0.0 [0.0–3.5]	0.0 [0.0–3.5]	0.0 [0.0–4.0]	1.0 [1.0–1.0]
Hypertension	0.5 [0.0–1.0]	0.0 [0.0–1.5]	0.0 [0.0–1.5]	0.0 [0.0–1.5]	1.0 [1.0–1.75]
Ischemic Heart Disease	1.0 [0.0–2.0]	0.0 [0.0–4.0]	0.0 [0.0–5.0]	0.0 [0.0–5.0]	1.0 [1.0–2.0]
Other/Multiple	2.0 [2.0–2.0]	4.0 [4.0–4.0]	4.0 [4.0–4.0]	5.0 [5.0–5.0]	1.0 [1.0–1.0]

Legend: Distribution of median satisfaction scores and interquartile ranges (IQR) for five core dimensions of rehabilitation experience, stratified by primary chronic condition. Scores are based on Likert-type items ranging from 1 (very poor) to 5 (very good), except for Difficulties in Access, which used a reversed scoring scale. Data are reported as median [IQR]. A higher score generally indicates a more favorable evaluation.

**Table 6:** Thematic Analysis of Open-Ended Responses.

Theme	Frequency
Lack of public services/coverage	38
Cost-related barriers	15
Insufficient referral/lack of information	5
Availability in rural areas	4
Home-based service needs	3

Legend: Summary of key themes identified from qualitative responses to the open-ended survey item on post-COVID-19 rehabilitation. Only responses that contained analyzable content (n = 65) were included in the thematic analysis. Themes were assigned based on recurring patterns related to access barriers, information gaps, and service delivery preferences. Frequencies represent the number of participants who mentioned each theme. Participants could refer to more than one theme in their responses.

## Data Availability

The data presented in this study are not publicly available due to ethical and legal considerations related to participant confidentiality. However, de-identified datasets may be made available from the corresponding author and principal investigator upon reasonable request.

## Supplementary Materials

The following supporting information can be downloaded at https://www.mdpi.com/article/10.3390/healthcare13131532/s1, S1_Questionnaire_PostCOVID_Rehabilitation.docx.

## Abbreviations

The following abbreviations are used in this manuscript:

AI	Artificial Intelligence
COPD	Chronic Obstructive Pulmonary Disease
COVID-19	Coronavirus Disease 2019
GDPR	General Data Protection Regulation
GenAI	Generative Artificial Intelligence
IQR	Interquartile Range
IRB	Institutional Review Board
MDPI	Multidisciplinary Digital Publishing Institute
NICE	National Institute for Health and Care Excellence
SARS-CoV-2	Severe Acute Respiratory Syndrome Coronavirus 2
SD	Standard Deviation
WHO	World Health Organization
