# Supplementary material for: Perceptions of Rehabilitation Access After SARS-CoV-2 Infection in Romanian Patients with Chronic Diseases: A Mixed-Methods Exploratory Study
Source: Healthcare (Basel). 2025 Jun 27;13(13):1532. doi: 10.3390/healthcare13131532 (PMC12249004; doi:10.3390/healthcare13131532)
Supplement: Supplementary file 1 [file healthcare-13-01532-s001.zip › healthcare-3671136-supplementary.pdf]

## Questionnaire on Patient Perceptions of Access and Quality of Post-COVID-19 Rehabilitation Services in Romanian Individuals with Chronic Diseases

---

**Study title:**
**Perceptions of Rehabilitation Access after SARS-CoV-2 Infection in Romanian Patients with Chronic Diseases: A Mixed-Methods Exploratory Study**

**Purpose:**
To evaluate patient perceptions regarding access to and quality of post-COVID-19 rehabilitation services, with a focus on those living with chronic comorbidities.

**Estimated completion time:** 5–7 minutes

**Note:** This questionnaire was self-developed by the research team. Although it was not previously validated through formal psychometric testing, its structure was based on the study objectives, prior research, and expert knowledge of the Romanian healthcare system.

---

### Section I – General Information

1. **Age:** _______ years
2. **Gender:** ☐ Male ☐ Female
3. **Place of residence:** ☐ Urban ☐ Rural
4. **Education level:**
   ☐ Primary ☐ Secondary ☐ High school ☐ Higher education
5. **Chronic conditions (check all that apply):**
   ☐ Hypertension ☐ Diabetes ☐ Cancer ☐ Ischemic heart disease
   ☐ COPD ☐ Other: ______________________

---

### Section II – COVID-19 Infection History

6. Were you hospitalized due to COVID-19?
   ☐ Yes ☐ No
7. How long has it been since your COVID-19 infection?
   ☐ <3 months ☐ 3–6 months ☐ 6–12 months ☐ >1 year
8. How severe was your COVID-19 illness?
   ☐ Mild ☐ Moderate ☐ Severe ☐ Very severe (ICU)

---

## Section III – Access to Rehabilitation Services

9. Have you received any post-COVID-19 rehabilitation services?
   ☐ Yes ☐ No
10. If yes, how soon after discharge did you begin rehabilitation?
    ☐ <1 month ☐ 1–3 months ☐ 3–6 months ☐ >6 months
11. Where did you receive rehabilitation services?
    ☐ Public hospital ☐ Private clinic ☐ At home ☐ Did not receive any
12. What types of rehabilitation services did you receive? (check all that apply):
    ☐ Kinetotherapy
    ☐ Physiotherapy
    ☐ Psychological counseling
    ☐ Respiratory rehabilitation
    ☐ Nutritional counseling
    ☐ Other: ______________________

---

## Section IV – Evaluation of Received Services

**(Please rate on a scale of 1 = very dissatisfied to 5 = very satisfied)**

13. Satisfaction with access to services:
    ☐ 1 ☐ 2 ☐ 3 ☐ 4 ☐ 5
14. Satisfaction with quality of services:
    ☐ 1 ☐ 2 ☐ 3 ☐ 4 ☐ 5
15. Did the staff show empathy and professionalism?
    ☐ 1 ☐ 2 ☐ 3 ☐ 4 ☐ 5
16. Did the rehabilitation improve your general health status?
    ☐ 1 ☐ 2 ☐ 3 ☐ 4 ☐ 5
17. Did you face difficulties in accessing services (e.g., waiting lists, costs, distance)?
    ☐ 1 ☐ 2 ☐ 3 ☐ 4 ☐ 5

---

## Section V – Suggestions

18. What improvements would you suggest for post-COVID-19 rehabilitation services in Romania?

    ________________________________________________________
    ________________________________________________________
